# On the Treatment of Ununited Fracture

**Published:** 1852-03

**Authors:** Daniel Brainard

**Affiliations:** Prof. of Surgery in Rush Medical College, etc.; Chicago


### ARTICLE VII.

*On the Treatment of Ununited Fracture by sub-cutaneous perforation of the bone, with a Case* By DANIEL BRAINARD, M. D., Prof. of Surgery in Rush Medical College.

No one acquainted with either the literature or the practice of Surgery will doubt that our knowledge of the treatment of false joint is extremely imperfect. The published cases and statistical tables prove this; but it is rendered still more evident by the far greater number of uncured and unrecorded cases to be met with in practice, in the more remote sections of our country. These but a small proportion of which fall under the notice of a single person, would form if they could be collected together an array calculated to weaken our confidence in the means usually resorted to for their cure. I have been led to this conclusion from having during the last fourteen years met almost yearly with one or more cases of the kind and from having found that they present themselves to, such of my professional friends as I have had opportunity of consulting on the subject.

These cases are in this country almost invariably treated by the seaton and when this is unsuccessful they are given up as hopeless. Of four cases which have presented themselves to my observation during the current year two were of that kind. One of these of the arm was of eleven years standing, the seaton had been used a year without benefit; the other will be given in detail.

It is greatly to be regretted that Dr. Physic whose experience with the seaton must have been extensive, did not have a record of its results. It is asserted on the one hand he lost confidence in it. This is denied on the other hand by Norris, who had every means of information, who says: "We have authority for stating that up to the period of his death, Dr. Physic *always* advocated the treatment of these cases by the seaton." * Nevertheless, we find in the same article, that " The caustic potash has been successfully used in three or four cases by Dr. J. R. Barton of this city in one of which" of the leg, "Dr. Physic discouraged the use of the seaton for fear of its failure." Gibson also states, that Dr. Physic discouraged its use in cases of fracture of the femur.

I make these remarks not from a disposition to undervalue the seaton, which is probably the most useful means yet known but to show that it is not universally successful or applicable to all cases. Dr. Norris in the article referred to, which is the best source of information to which I could refer, says that, "in the femur it has often failed." I was myself in the habit until about four years ago of abandoning most cases in which the seaton had failed not choosing to resort to resection or amputation. In 1848 I treated a case successfully by passing a silver wire around the ends of the fragments. Further reflections suggested different modes of treatment which I have applied.

A little reflection will, with the aid of our present knowledge of pathology, convince us that the seaton is not calculated to be generally successful.

---

* Amer. Jour. Med. Science, Vol. 3, New Series, pp. 55.

The first condition most favorable for the production of callus is the effusion of a *blastema* which should not be converted into pus and dischargal.

The second is, that this blastema should be in contact with a freshly wounded surface of bone—the law of "analagous formation" holding good here.

"The blastema between areolar tissue becomes areolar tis- at the extremity of divided nerves it forms nervous substance, &c., (Vogel.)

Now the seaton necessarily causes the convertion of the effused blastema into pus while its introduction produces no freshly wounded surface of bone; the case is converted into a compound fracture where it was desirable to only re-produce a simple one.

Can such cutaneous wounds of bones be made without danger of seppuration? To determine this point I at different times perforated all the principal bones of the members of a dog and did not find that suppuration in any case resulted.

Case treated by perforation of the ends of the bones—

Allcett Barnes, aet. 26 years, received June 10th, 1850, a simple fracture from being carried by a belt around a shaft. It was dressed by Dr. Hawley of Yorkville, Mich., two splints being properly applied. The dressing was changed perhaps once a week for eight weeks when the ulna was found to be united but the radius was not. A simple bandage was placed about it for four weeks when he consulted Dr. White of Kalamazoo, who put on carved splints for a month, when finding no sign of union he put through a skein of silk for a seaton which was allowed to remain three weeks. It caused much pain and suppuration. When the seaton was taken out a bandage was put about it for a week, when splints were applied and continued five weeks. They were then taken off and no union found to have taken place. Such was the account given by the patient himself.

Feb. 4th, 1851. Ununited fracture of the radius found above one third of the distance from the wrist to the elbow, partially overlapped, oblique, moveable, and the hand of little use.

*Operation.*—Having provided several *brad awls,* such as are used by shoe-makers, and had them well tempered and tried on dry bones, I carried one of them through the skin opposite the fracture and by movements of partial rotation perforated both fragments where they overlapped. The awl being then withdrawn from the bone, (but not from the skin,) was directed obliquely upward, then obliquely downwards so as to make three perforations. It was then entirely withdrawn, and collodion put upon the puncture of the skin. The member was dressed with the immoveable apparatus. Some tenderness was the effect.

Feb. 17th. The tenderness having subsided I removed the bandage, repeated the operation in the same manner, choosing a different point of puncture and re-applied the dressing.

March 11th. Repeated the operation again and dressed as before, mobility scarcely perceptible.

March 21st. Dressings removed, union perfect.

The dressings were in this case continued on near seven weeks, but it is probable the last perforation and dressing might well have been omitted.

The occurrence of union in so short a time where the seaton had failed and with no operation which interfered with the comfort or amusement of the patient, was a most favorable result, but not different from what was anticipated. I do not know that this sub-cutaneous perforation had been used by any one. Perforations by incision and inserting pieces of metal have been tried. I myself treated a case in this manner. It was on a man 36 years of age, who had long deposited phosphatic gravel in the urine, and in his youth been affected with necrosis of the femur. It was near the middle of the femur, and the fragments extremely moveable.

Sept. 20, 1850. I made an inscision an inch in length over the false joint and down to the bone. I then perforated the extremity of each fragment with a *bitt*, and putting in a director from a pocket case dressed the limb in the angular apparatus for the lower extremity. The director was taken out in about ten days.

In this case the dressing was removed in eleven weeks and the patient was able to walk at the end of four months. At the present time (Jan. 1852,) he is pursuing an active occupation with a good limb.

Notwithstanding the favorable result of this case it must be admitted that the symptoms were severe, like those resulting from resection. Such were they also in the case treated by the wire, so that although it seems probable that in both these cases the result was at least as favorable as could be expected from resation, it is also true that the danger incurred is not materially less.

In all these cases the operation is severe, while the sub-cutaneous perforation is free from danger and reasoning from the analogy of simple fractures, or judging from the result of a single case, promises to be much more efficient.

*Chicago, Jan.* 25, 1852.

